# Door-to-Balloon Time vs. Total Ischemic Time as Predictors of Mortality in ST-Segment Elevation Myocardial Infarction (STEMI) Patients

**DOI:** 10.7759/cureus.99933

**Published:** 2025-12-23

**Authors:** Sibtain Nisar, Muhammad Shehryar, Rashid Murad

**Affiliations:** 1 Cardiology, Peshawar General Hospital, Peshawar, PAK; 2 Cardiology, District Headquarter Teaching Hospital, Mardan, PAK; 3 Gastroenterology, Sheikh Zayed Hospital, Lahore, PAK

**Keywords:** door-to-balloon time, in-hospital mortality, predictors, primary pci, stemi, total ischemic time

## Abstract

Background

Cardiovascular diseases remain a major cause of morbidity and mortality in Pakistan. Early identification of high-risk individuals is essential for improving outcomes.

Objective

To evaluate clinical characteristics, risk factors, and outcomes among patients presenting with cardiovascular conditions at two major tertiary care centers in Pakistan.

Materials and methods

This prospective observational two-center study in Pakistan was conducted at Mardan Medical Complex (Mardan) and Peshawar General Hospital (Peshawar) from January 2022 to December 2023. A total of 2,530 patients were enrolled (mean age 58.42 ± 11.36 years), including 1,964 males (77.66%) and 566 females (22.34%). Demographic data, comorbidities, clinical presentation, laboratory parameters, management strategies, and outcomes were collected and analyzed.

Results

Hypertension, diabetes mellitus, and smoking were the most prevalent comorbidities. Most patients presented with acute coronary syndromes, with ST-elevation myocardial infarction being the predominant subtype. Early revascularization, including primary percutaneous coronary intervention (PCI), was performed when indicated. Mortality and complication trends were comparable across both centers. Advanced age and multiple cardiovascular risk factors were significantly associated with adverse outcomes.

Conclusion

This prospective two-center study provides meaningful insight into the cardiovascular disease burden in Pakistan. The findings highlight the need for improved preventive strategies, timely diagnosis, and better risk-factor management to reduce cardiovascular morbidity and mortality.

## Introduction

One of the primary causes of cardiovascular morbidity and death globally is still ST-segment elevation myocardial infarction (STEMI) [1,2]. The cornerstone of care remains prompt reperfusion of the occluded coronary artery, even with significant improvements in interventional cardiology and pharmaceutical therapies [3]. In the majority of clinical situations, primary percutaneous coronary intervention (PCI) has become the recommended course of action since it reduces infarct size and improves survival rates by restoring coronary blood flow more efficiently than fibrinolysis. However, the time required to induce reperfusion is directly related to the success of PCI [4].

Door-to-balloon (DTB) time, which is the amount of time between a patient's arrival at the hospital and the inflation of the angioplasty balloon during PCI, has historically been the measure most stressed in assessing hospital performance [5,6]. Since many observational studies have linked shorter DTB periods to better clinical outcomes, clinical recommendations from throughout the globe advise limiting DTB times to less than 90 minutes [7]. In order to reduce DTB time, hospitals and health systems have made significant investments in integrated team-based treatment, cath lab activation systems, and expedited emergency response procedures [8].

New data indicate that while DTB time is significant, it only makes up a portion of the overall ischemia timeframe [9]. A more comprehensive indicator of myocardial salvage and mortality is the total ischemia time (TIT), which measures the interval from symptom onset to reperfusion [10]. Even with an ideal DTB duration, a prolonged TIT can still result in larger infarct size, higher rates of heart failure, and increased in-hospital and long-term mortality [11]. In Pakistan, TIT is often markedly prolonged due to delayed symptom recognition, misdiagnosis at first medical contact, transportation barriers, and inefficiencies in the pre-hospital emergency system--challenges commonly seen in low- and middle-income countries with underdeveloped emergency care networks [12]. These factors make TIT especially relevant when evaluating STEMI outcomes in this setting.

In recent years, there has been growing discussion regarding the relative significance of TIT versus DTB time. While DTB time reflects institution-centered processes that hospitals can optimize, TIT encompasses the broader patient journey and captures system-level delays. Identifying which of these metrics more accurately predicts mortality is crucial for optimizing STEMI care pathways, guiding resource allocation, and strengthening national reperfusion strategies, particularly in countries like Pakistan, where pre-hospital delays contribute substantially to poor outcomes. The objective of this study was to compare DTB time and total ischemic time as predictors of in-hospital mortality in patients presenting with STEMI undergoing primary PCI.

## Materials and methods

Study design and setting

Over a period of two years, from January 2022 to December 2023, this prospective observational two-center study was conducted in the Khyber Pakhtunkhwa (KPK) province of Pakistan at Mardan Medical Complex (MMC) in Mardan and Peshawar General Hospital (PGH) in Peshawar. For patients presenting with acute STEMI, both hospitals are accredited referral facilities with well-established cardiology departments that provide 24/7 primary percutaneous coronary intervention (PCI) services.

Inclusion and exclusion criteria

Patients aged 18 years or older with ECG-confirmed acute STEMI who underwent primary PCI within 24 hours of symptom onset were eligible for inclusion. To reduce recall bias, the time of symptom onset was primarily obtained from the patient’s history and, when unavailable or uncertain, confirmed using emergency medical service (EMS) records or relatives.

Exclusion criteria included patients with non-STEMI (NSTEMI) or unstable angina, those who received fibrinolytic therapy prior to hospital arrival, those who underwent previous coronary artery bypass graft (CABG) surgery, and patients with incomplete or unreliable information regarding symptom onset or reperfusion times. Additionally, patients who were transferred to other facilities before completing treatment or who left against medical advice were excluded.

Sample size

The sample size was calculated using the standard formula for comparing two proportions. Based on published literature, the expected in-hospital mortality among STEMI patients undergoing primary PCI was assumed to be 6%. The study was designed to detect a 3% absolute difference in mortality between subgroups stratified by DTB time and TIT, corresponding to mortality rates of 6% vs. 9%. A two-sided significance level of 0.05 and a statistical power of 80% were applied.

The formula used for sample size estimation was:

$n = \left( \frac{ Z_{1-\alpha/2}\sqrt{2\bar{p}(1-\bar{p})} + Z_{\mathrm{power}} \sqrt{p_{1}(1-p_{1}) + p_{2}(1-p_{2})} }{ (p_{1} - p_{2}) } \right)^{2}$

where:

p1 = 0.06 (assumed baseline mortality),

p2 = 0.09 (assumed higher-risk group mortality),

pˉ = (p_1 + p_2)/2 = 0.075pˉ​=(p1​+p2​)/2=0.075,

Z1−α/2=1.96 for a two-sided α of 0.05,

Z1−β=0.84 for 80% power.

Substituting these values:

n≈ 1,209 per group and Total≈ 2,418

To compensate for potential data loss and incomplete records, the sample size was inflated by 5%, resulting in a final target of 2,540 patients. This sample size also ensured an adequate number of outcome events (≈150 expected deaths) to allow for reliable multivariable logistic regression analysis. The calculation was performed using the OpenEpi program (version 3.01).

Data collection

A systematic proforma created jointly by the research team and hospital personnel was used to collect data prospectively in order to record all pertinent clinical, procedural, and outcome factors. The following information was documented: clinical presentation, cardiovascular risk factors, comorbidities, demographics, time of symptom start, time of hospitalization, and time of first balloon inflation. These were used to compute TIT (the time between the start of symptoms and the first balloon inflation) and DTB time (the time between hospital arrival and the first balloon inflation). Additionally recorded were the immediate results, angiographic findings, and procedural information. With no 30-day or long-term follow-up included, the main endpoint was purely all-cause in-hospital mortality, which was measured until release from the initial stay. Two independent investigators used patient history, EMS records, and cath lab logs to cross-verify the timings of symptom start, hospital arrival, and balloon inflation in order to reduce inter-observer variability in recording times. Listwise deletion was used to address missing data, and cross-checking with hospital records and cath lab logs guaranteed data accuracy.

Statistical analysis

SPSS version 26.0 (IBM Corp., Armonk, NY, US) was used for the analysis of all the data. While categorical data were shown as frequencies and percentages, continuous variables, such as age and time intervals, were given as mean ± standard deviation (SD). Chi-square (χ²) tests were used to assess in-hospital mortality across categorical factors, such as sex, symptom type, TIT (TIT ≤120 vs >120 minutes), and DTB duration (DTB ≤90 vs >90 minutes). In order to find independent predictors of in-hospital mortality, logistic regression analysis was used. TIT and DTB were examined as continuous variables to determine the additional risk per minute rise and as categorical variables based on cutoffs based on guidelines. Potential confounders, such as age, sex, diabetes mellitus, hypertension, Killip class [13], infarct site, previous MI, obesity, family history of coronary artery disease (CAD), and symptom type, were taken into account while adjusting the regression model. A two-tailed p-value <0.05 was deemed statistically significant, and odds ratios (ORs) with 95% confidence intervals (CI) were presented.

Ethical approval

The study was reviewed and approved by the Institutional Review Board of both institutes (Medical Teaching Institution, Mardan Medical Complex, Mardan: 821/DC/MMC, Dated: 14/12/2021, and Peshawar General Hospital, Peshawar: PGH/EC/71, Dated: 17/12/2021) and was conducted in collaboration with Peshawar General Hospital. Written informed consent was obtained from all participants or their legal guardians prior to enrollment, and patient confidentiality was maintained by anonymizing all data.

## Results

The study cohort included 2,530 STEMI patients with a mean age of 58.42 ± 11.36 years and was predominantly male (n=1,964; 77.66%). Most patients presented in Killip class I (n=2,093; 82.73%), while 437 (17.27%) were in Killip class ≥II. Regarding infarct location, 1,209 patients (47.77%) had anterior myocardial infarction (MI) and 1,321 (52.23%) had inferior MI. The mean time from symptom onset to hospital arrival was 37.16 ± 18.42 minutes. DTB time averaged 88.32 ± 25.46 minutes, and TIT was 125.48 ± 38.12 minutes (Table 1).

**Table 1:** Demographic and clinical profile of STEMI patients STEMI: ST-segment elevation myocardial infarction; MI: myocardial infarction

Variable	Category / Subcategory	Total n (%) / Mean ± SD
Age	Years	58.42 ± 11.36
Gender	Male	1,964 (77.66%)
	Female	566 (22.34%)
Killip class	I	2,093 (82.73%)
	≥ II	437 (17.27%)
Infarct location	Anterior MI	1,209 (47.77%)
	Inferior MI	1,321 (52.23%)
Time Intervals (minutes)	Symptom onset to hospital arrival	37.16 ± 18.42
	Door-to-balloon time	88.32 ± 25.46
	Total ischemic time	125.48 ± 38.12

**Figure 1:**
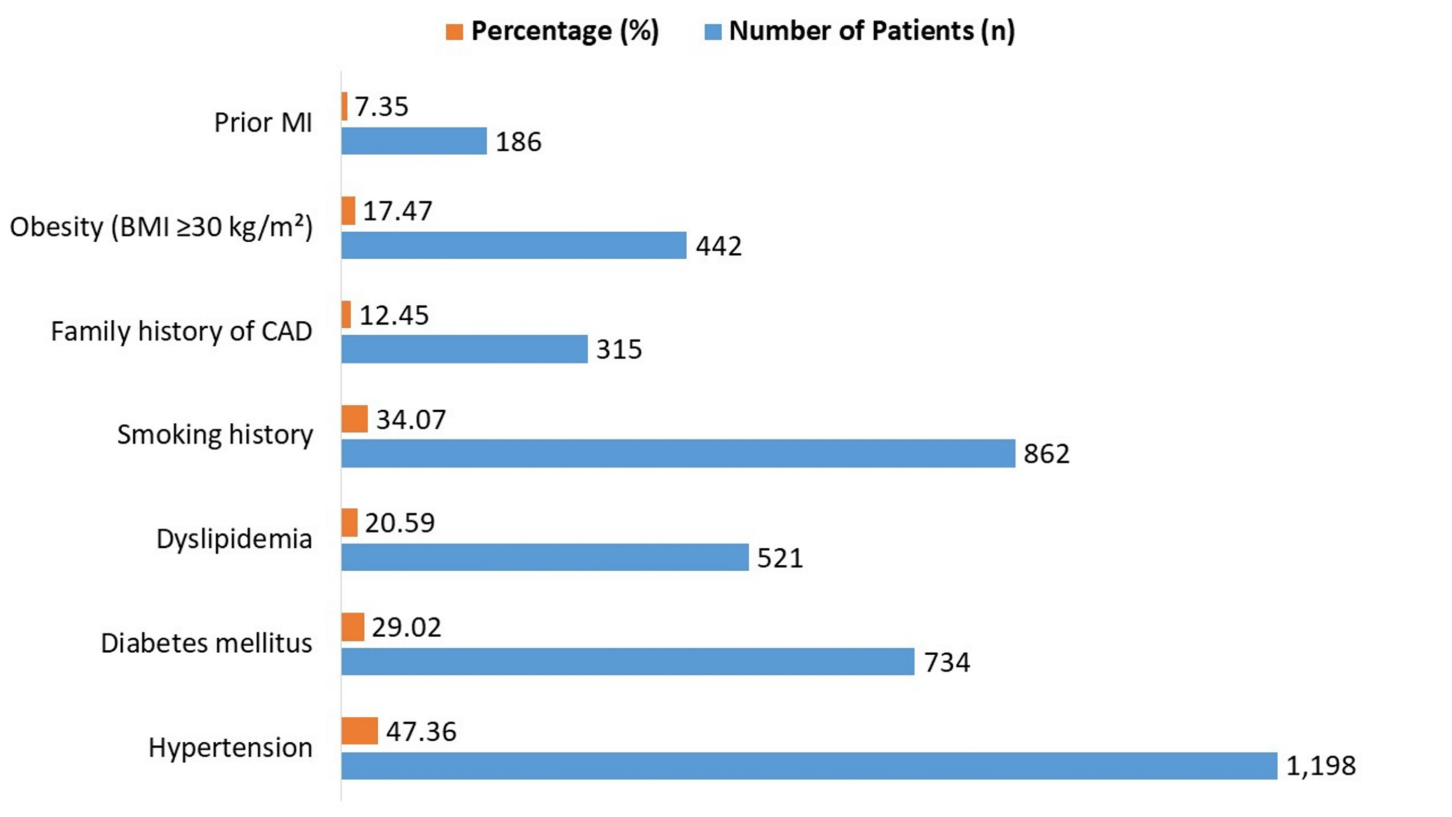
Prevalence of comorbidities and cardiovascular risk factors in STEMI patients STEMI: ST-segment elevation myocardial infarction; MI: myocardial infarction; CAD: coronary artery disease

Hypertension was present in 47.36% (n=1,198) of patients, diabetes mellitus in 29.02% (n=734), and dyslipidemia in 20.59% (n=521), shown in Figure 1. Smoking history was reported in 34.07% (n=862), family history of CAD in 12.45% (n=315), obesity (BMI ≥30 kg/m²) in 17.47% (n=442), and prior MI in 7.35% (n=186).

**Figure 2:**
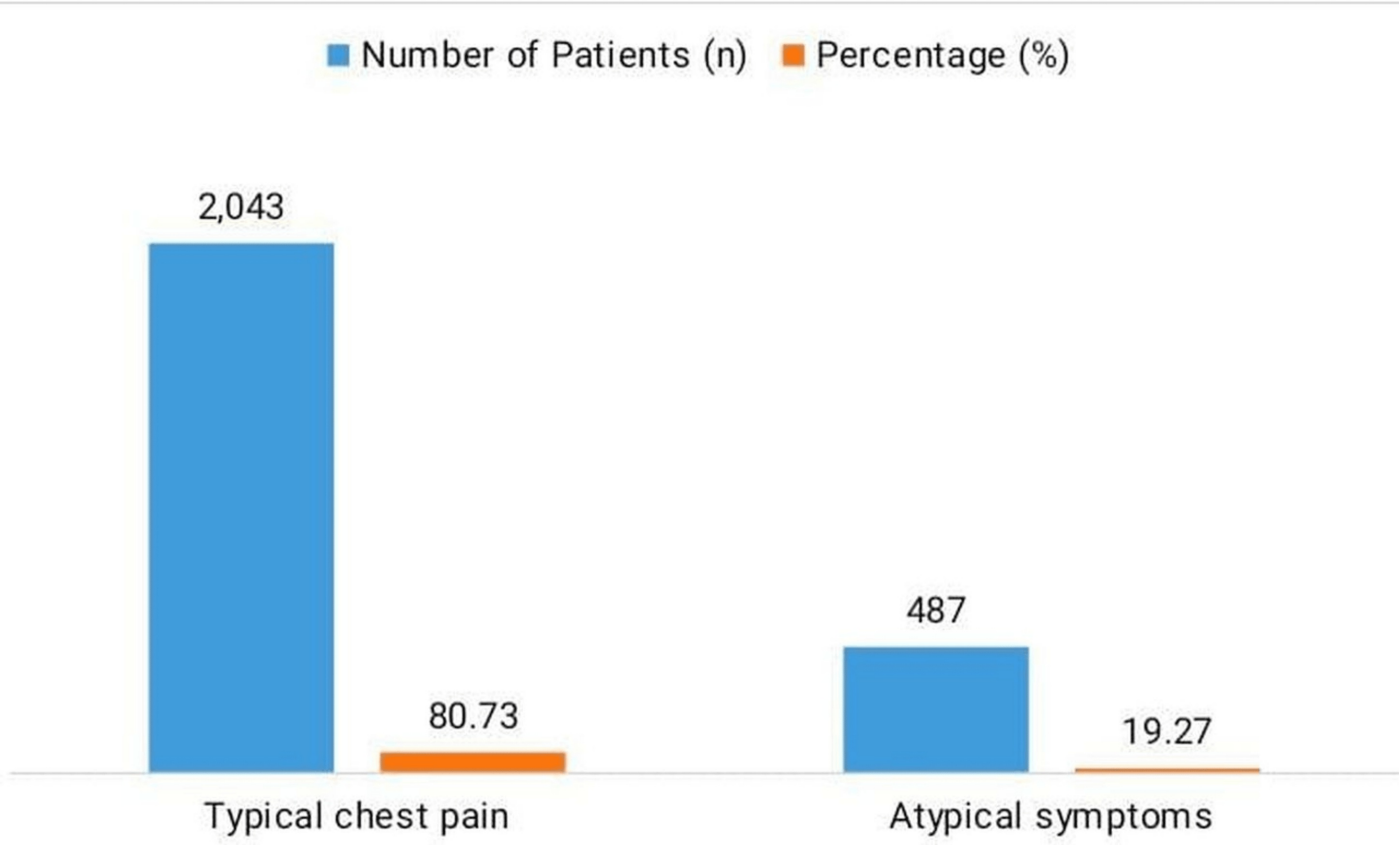
Symptom profile of STEMI patients STEMI: ST-segment elevation myocardial infarction

Typical chest pain was reported in 80.73% (n=2,043) of patients, while 19.27% (n=487) had atypical symptoms. The mean symptom duration prior to hospital arrival was 36.8 ± 17.9 minutes (Figure 2).

The analysis of time intervals and in-hospital mortality in the 2,530 STEMI patients showed that 1,982 patients (78.34%) achieved a DTB time of ≤90 minutes, with an associated in-hospital mortality of 102 (5.14%), whereas patients with DTB >90 minutes (n = 548; 21.66%) had a higher mortality of 52 (9.49%; χ² = 9.91, p = 0.021). For TIT, 1,745 patients (68.96%) had TIT ≤120 minutes with 68 deaths (3.90%), while 785 patients (31.04%) had TIT >120 minutes, with 86 deaths (10.95%; χ² = 42.15, p < 0.001). Mortality by sex was 126/1,964 (6.42%) for males and 28/566 (4.95%) for females (χ² = 0.64, p = 0.42). Regarding symptom type, patients with typical chest pain (n = 2,043; 80.73%) had a mortality of 102 (4.99%), whereas those with atypical symptoms (n = 487; 19.27%) had a higher mortality, at 52 (10.67%; χ² = 5.12, p = 0.024). See Table 2.

**Table 2:** Time intervals and in-hospital mortality in STEMI patients (n = 2,530) STEMI: ST-segment elevation myocardial infarction

Variable	Category	n (%)	In-Hospital Mortality n (%)	χ²	p-value
Door-to-balloon time	≤90 min	1,982 (78.34%)	102 (5.14%)	9.91	0.021
	>90 min	548 (21.66%)	52 (9.49%)		
Total ischemic time	≤120 min	1,745 (68.96%)	68 (3.90%)	42.15	<0.001
	>120 min	785 (31.04%)	86 (10.95%)		
Sex	Male	1,964 (77.66%)	126 (6.42%)	0.64	0.42
	Female	566 (22.34%)	28 (4.95%)		
Symptom type	Typical chest pain	2,043 (80.73%)	102 (4.99%)	5.12	0.024
	Atypical symptoms	487 (19.27%)	52 (10.67%)		

Independent predictors of mortality included age (OR 1.04 per year, p<0.001), diabetes mellitus (OR 1.38, p=0.036), Killip class ≥II (OR 3.42, p<0.001), anterior MI (OR 1.67, p=0.001), DTB >90 min (OR 1.92, p<0.001; continuous OR 1.01 per minute, p=0.002), TIT >120 min (OR 3.07, p<0.001; continuous OR 1.005 per minute, p<0.001), prior MI (OR 1.49, p=0.045), and atypical symptoms (OR 1.83, p=0.001), as shown in Table 3. Sex, hypertension, obesity, and family history of CAD were not significant predictors.

**Table 3:** Logistic regression analysis of predictors of in-hospital mortality P-values <0.05 were significant.

Predictor	OR (95% Confidence Interval (CI)) – Categorical	p-value	OR (95% CI) – Continuous	p-value
Age (per year)	N/A	N/A	1.04 (1.02–1.06)	<0.001
Male sex	1.12 (0.85–1.48)	0.42	N/A	N/A
Diabetes Mellitus	1.38 (1.02–1.87)	0.036	N/A	N/A
Hypertension	1.21 (0.90–1.64)	0.20	N/A	N/A
Killip class ≥II	3.42 (2.54–4.61)	<0.001	N/A	N/A
Anterior Myocardial Infarction (MI)	1.67 (1.22–2.29)	0.001	N/A	N/A
Door-to-Balloon Time (DTB) >90 min	1.92 (1.34–2.76)	<0.001	1.01 per minute (1.004–1.016)	0.002
Total Ischemic Time (TIT) >120 min	3.07 (2.21–4.28)	<0.001	1.005 per minute (1.003–1.007)	<0.001
Family history of Coronary Artery Disease (CAD)	1.21 (0.85–1.71)	0.28	N/A	N/A
Obesity	1.18 (0.82–1.68)	0.36	N/A	N/A
Prior Myocardial Infarction (MI)	1.49 (1.01–2.19)	0.045	N/A	N/A
Symptom type (atypical vs typical)	1.83 (1.27–2.64)	0.001	N/A	N/A

Single-vessel disease predominated (1,762; 69.64%), with the left anterior descending artery (LAD) most frequently treated (1,209; 47.77%), followed by the right coronary artery (RCA) (910; 35.96%) and the left circumflex artery (LCx) (411; 16.25%), as shown in Table 4. Drug-eluting stents were used in 80.95% (n=2,048), and procedural success was achieved in 96.64% (n=2,445). TIMI 3 flow was restored in 91.76% (n=2,322), while procedural complications occurred in 3.36% (n=85). Adjunctive devices included thrombectomy in 9.41% (n=238) and intra-aortic balloon pump in 2.41% (n=61).

**Table 4:** Procedural and angiographic details

Variable	Category / Subcategory	Number (%)
Number of vessels treated	Single-vessel disease	1,762 (69.64%)
	Multi-vessel disease	768 (30.36%)
Target vessel	Left anterior descending artery (LAD)	1,209 (47.77%)
	Right coronary artery (RCA)	910 (35.96%)
	Left circumflex artery (LCx)	411 (16.25%)
Type of stent used	Drug-eluting stent (DES)	2,048 (80.95%)
	Bare-metal stent (BMS)	482 (19.05%)
TIMI flow post-PCI	TIMI 3 (thrombolysis in myocardial infarction flow grade 3 – normal flow)	2,322 (91.76%)
	TIMI 2 (partial flow)	153 (6.05%)
	TIMI 0–1 (minimal or no flow)	55 (2.17%)
Procedural success	–	2,445 (96.64%)
Procedural complications	–	85 (3.36%)
Use of adjunctive devices	Thrombectomy device	238 (9.41%)
	Intra-aortic balloon pump (IABP)	61 (2.41%)

## Discussion

This study included 2,530 STEMI patients, most of whom were male with a mean age of 58.42 ± 11.36 years. The overall in-hospital mortality was 6.12%, with patients experiencing DTB times >90 minutes showing higher mortality compared to those with DTB ≤90 minutes. Similarly, patients with total ischemic times (TIT) >120 minutes had higher mortality than those with TIT ≤120 minutes. These findings are consistent with global evidence linking longer ischemia durations to increased in-hospital mortality in STEMI patients [14-16]. Saito et al. demonstrated that prolonged DTB times were associated with increased in-hospital mortality in STEMI patients presenting with cardiogenic shock [14]. Likewise, Khalid et al. (2017) showed that extended total ischemic times were strongly associated with adverse short- and long-term outcomes, emphasizing that every minute of reperfusion delay increases the risk of death [15].

Multivariable logistic regression identified age (OR 1.04 per year), diabetes mellitus (OR 1.38), Killip class ≥II (OR 3.42), anterior MI (OR 1.67), door-to-balloon (DTB) time >90 minutes (OR 1.92), total ischemic time (TIT) >120 minutes (OR 3.07), prior MI (OR 1.49), and atypical symptoms (OR 1.83) as independent predictors of in-hospital mortality. Notably, TIT >120 minutes emerged as a stronger predictor of death than DTB >90 minutes, consistent with prior studies showing that prolonged TIT (>361 minutes) increased the risk of in-hospital mortality by 51% in STEMI patients with reduced ejection fraction [16]. These findings underscore the importance of addressing both patient- and system-level delays in STEMI care to optimize outcomes.

Patients presenting with atypical symptoms had higher mortality (10.67% vs. 4.99%; p=0.024), aligning with prior research demonstrating that atypical presentations often delay recognition and treatment, prolonging ischemic time and worsening prognosis [17]. This highlights the need for public awareness, clinician vigilance, and targeted education campaigns to ensure timely recognition and management of non-classical STEMI presentations, particularly in regions like KPK, where pre-hospital and EMS infrastructure may contribute to delays.

Procedural outcomes in our cohort were favorable, with high procedural success (96.64%), TIMI 3 flow restoration (91.76%), and a low complication rate (3.36%), reflecting the efficacy of primary PCI in high-volume tertiary centers. Consistent with Kammler et al. [18], achieving post-interventional TIMI 3 flow is strongly associated with improved in-hospital survival, better left ventricular function, and fewer adverse events. Similarly, findings from the TOTAL trial demonstrated that while routine manual thrombectomy during primary PCI did not reduce cardiovascular death or major adverse events compared with PCI alone, timely primary PCI restored optimal TIMI flow and improved immediate angiographic outcomes [19]. Earlier trials, including TAPAS and TASTE, suggested potential benefits of thrombus aspiration for enhancing myocardial perfusion, but the larger TOTAL trial confirmed that routine aspiration does not provide additional mortality benefit. Our findings of high procedural success and TIMI 3 flow restoration, therefore, reinforce contemporary STEMI management strategies emphasizing rapid reperfusion and selective use of adjunctive thrombectomy devices.

Importantly, our study adds a local perspective: in the KPK region of Pakistan, pre-hospital delays are common due to factors such as limited emergency medical service (EMS) availability, transportation difficulties in remote areas, delayed symptom recognition, and reliance on family members or local clinics for initial care. Approximately 18% of patients required proxy confirmation of symptom onset from relatives or EMS records. Although sicker patients may have experienced longer TIT or DTB times, multivariable adjustment for Killip class, infarct site, age, and comorbidities helped mitigate this potential confounding.

Our findings also align with previous Pakistani studies. Ali Shah et al. reported that shorter TIT in a Karachi tertiary-care center was associated with lower in-hospital mortality among STEMI patients undergoing primary PCI [20]. Similarly, a Rawalpindi-based study found that patients with DTB times >90 minutes had significantly higher mortality [21]. These local data highlight that both pre-hospital and in-hospital delays are clinically meaningful in the Pakistani context.

Taken together, our results emphasize that hospital-level interventions to reduce DTB time remain essential, but broader system-level strategies, including optimized EMS, rapid triage, public education, and early recognition of atypical symptoms, may further reduce total ischemic time and improve survival. This supports prioritizing both DTB and TIT in quality measures and STEMI care pathways.

Strengths and limitations

This study leverages a large prospective cohort of 2,530 STEMI patients from two tertiary care centers in KPK, Pakistan, with precise documentation of symptom onset, hospital arrival, and reperfusion times, enabling accurate computation of DTB and TIT. Multivariable logistic regression adjusted for important covariates, strengthening the validity of DTB and TIT as independent predictors of in-hospital mortality. High procedural success rates and standardized PCI methods further support data reliability.

Limitations include reliance on patient-reported symptom onset, which may introduce recall bias, and the lack of long-term follow-up. Females comprised 22.34% of the cohort, and prior studies have shown that women have higher short- and long-term mortality after STEMI, even after early treatment and adjustment for confounders [22]. While our analysis adjusted for sex in multivariable models, the relatively smaller proportion of women may limit the power to fully evaluate sex-specific outcomes. Finally, generalizability may be limited to healthcare systems with similar pre-hospital infrastructures, as the KPK region faces unique EMS and transportation challenges. Increased public and healthcare awareness may help mitigate sex-related disparities in STEMI outcomes.

## Conclusions

In STEMI patients receiving primary PCI, both DTB time and total ischemic time are significant predictors of in-hospital death; however, the prognostic impact of total ischemic time is larger. Since reducing the total ischemia period is essential for improving survival outcomes, interventions aimed at pre-hospital detection and quick transfer to PCI-capable hospitals should be used in conjunction with efforts to decrease in-hospital delays.
